# Discovering down’s syndrome: An account from A low middle income country

**DOI:** 10.12669/pjms.40.9.9083

**Published:** 2024-10

**Authors:** Ayeza Ali, Nabiha Ali, Misbah Iqbal Hanif, Syed Rehan Ali

**Affiliations:** 1 Ayeza Ali, Liaquat National Hospital and Medical College, Karachi, Pakistan; 2 Nabiha Ali, Aga Khan University Hospital, Karachi, Pakistan; 3 Misbah Iqbal Hanif, Dow University of Health Sciences, Karachi, Pakistan. Sindh Institute of Child Health and Neonatology, Karachi, Pakistan; 4 Syed Rehan Ali, Sindh Institute of Child Health and Neonatology, Karachi, Pakistan

**Keywords:** Chromosomal abnormalities, Chromosomal numerical aberration, Down’s syndrome

## Abstract

**Objective:**

This study aims to establish the frequency of Down’s syndrome which will enhance the knowledge of our local population as well to understand our genotypic patterns and variations.

**Methods:**

Electronic Medical Records of inborn babies at the Department of Neonatology, Sheikh Saeed Memorial Campus of The Indus Hospital Karachi during the study period from 1^st^ January 2021 to 31^st^ December 2022 were retrieved. Chromosomal karyotyping was done for all babies with suspicious clinical features identified on routine new born examination by consultant neonatologists, trainee doctors and experienced nurses.

**Result:**

There was a total of 7,433 live births during the study period, out of which 14 babies had features suggestive of DS. repetition of sentence. What about karyotyping result??

**Conclusion:**

The frequency of DS in our study is slightly higher than the incidence reported within South East Asia. It is high time to perform effective antenatal screening and efficient prenatal diagnostic services for early detection of chromosomal numerical aberration such as Down syndrome for better management of upcoming pregnancies.

## INTRODUCTION

Down syndrome (DS) is the most common genetic syndrome with up to 1/800-1000 live births, caused by complete or partial addition of chromosome 21 (trisomy 21). Chromosomal aneuploidy (trisomy 21) arising from an abnormal cell division resulting in an extra copy of chromosome 21, due to the non-disjunction of chromosome during meiosis.^1^ This pan-ethnic condition has two cardinal features; hypotonia and intellectual disability. Both of these are present in all individuals with trisomy 21 in variable severity. Other common clinical presentations of DS include; sensorineural hearing defects (75%), obstructive sleep apnea (50-79%), otitis media (50-70%), eye problems including cataracts and refractive errors (60%), congenital heart diseases (50%), neurological problems (1-13%), gastrointestinal atresias (12%), endocrinological disorders including hypothyroidism (4-18%), blood disorders including transient myeloproliferative disorder (4-10%) and leukemia (1%) and hirchsprung disease (<1%).^2^

One of the foremost risk factor impacting the overall incidence of trisomy 21 is the conception at the advanced maternal age.^3^ The incidence increases drastically to 1 in 400 live births in those older than 35 years of age to as high as one in 12 live births by the age of 50 years. However, due to high pregnancy and birth rates the majority of trisomy 21 babies are born to mothers in the younger age group. Epidemiological studies reveal that with the present distribution of maternal ages, prenatal diagnosis among women 35 years and older can result in decrease in crude incidence of DS.^4^

The incidence however varies greatly with respect to ethnic back grounds and maternal age at the time of child birth, hence remaining high in various other parts of the world. Ni She R et. al.^5^, reports the incidence in Ireland to be the highest in Europe being one in 546 live births while another study from the Middle East reports it to be one in 449 live births. Moreover, in India it is 0.8 – 1.2/1000 live births while in China it is 0.3/1000 live births.^6^ Subsequently in UK it is one in 1000, USA one in 600^7^ Saudi Arabia one in 554 and Canada one in 740 live births or 13.5/10,000 live births.^8^

To the best of our knowledge, there are no local figures reporting the prevalence of DS from Pakistan. This study aims to establish the frequency of Down syndrome which will enhance the knowledge of our local figures to understand our genotypic patterns and variations. This will subsequently help us understand the genetic burden and work on the psychosocial impact of this disorder on our society.

## METHOD

This study was a retrospective cross-sectional analysis of new born infants with DS from 1^st^ January 2021 to 31^st^ December 2022. Electronic Medical Records of inborn babies at the Department of Neonatology, Sheikh Saeed Memorial Campus of The Indus Hospital Karachi during the study period were retrieved. Chromosomal karyotyping was done for all babies with suspicious clinical features identified on routine new born examination by consultant neonatologists, trainee doctors and experienced nurses. Screening for other associated congenital anomalies was also done. Samples for karyotyping and cytogenetic analysis were sent as the routine medical protocol. Babies with phenotypical features suggestive of other syndromes such as Trisomy 13 or 18 were excluded based on physical examination.

## RESULTS

There were 7,433 live births during the study period, out of which 14 babies had features suggestive of DS. Samples from all these babies were then sent to Agha Khan University Laboratory, Karachi for karyotyping and cytogenetic analysis after obtaining informed verbal consent from parents. Karyotyping was positive for DS in all 14 cases (100%) with meiotic non – disjunction being the most common genotypic variation in all cases with not a single evidence of mosaicism. There was a preponderance of females amongst the cases with male to female ratio of 1: 2.5. Mean maternal age at birth was 32 years (range 24 – 39 years) of which majority of mothers (42.8%) were 29 years of age or less while only 28.5% of mothers were >35 years of age. Most of the cases (64%) were products of non-consanguineous marriage. Family history was insignificant in majority of cases, none of the couple had other children with any other chromosomal anomaly. Over all we estimated the incidence of DS during study period to be 1 in 530 live births (1.8/1000 live births.

## DISCUSSION

Multiple studies on Down syndrome (DS) has yielded insights that extend well beyond the well-recognised primary characteristics of dysmorphism and intellectual impairment. Among a number of other characteristics that make up the DS phenotypical spectrum, such as congenital heart abnormalities, early ageing, Alzheimer’s disease, and childhood leukaemia, multisystem involvement is becoming more well acknowledged.

Guidelines for health supervision of DS individuals have been published by American Academy of Paediatrics which involves both screening and follow up of all associated congenital anomalies with DS from infancy to early adulthood. These include but are not limited to frequent auditory examination, monitoring for obstructive sleep apnoea, endocrinological problems like hypothyroidism, gastrointestinal associations like celiac disease and gastroesophageal reflux disease, haematological manifestations like leukemia, cardiovascular, ophthalmological, cognitive, behavioural and neurodevelopmental problems.^2^

The prevalence of Down syndrome in newborns varies according to region, age/group, race, ethnicity and gender. Our estimated DS prevalence at birth (1 in 530) included only live born infants with DS in one hospital. In comparison to the West, the estimated incidence is higher in our population. We obtained 100 results from karyotype analyses, confirming that our clinicians’ diagnostic approach is up to standard and aligns with previously reported findings in the literature.^8^ Our study also suggested that incidence of DS was higher among male babies than female, and the mean maternal age was 32. Majority (71.3%) of babies during our study period were born to mothers <35 years of age of which around 42% were born to mothers <29 years of age or less.^9^ This is most likely due to high birth rate amongst younger mothers, additionally 64% of them were products of non-consanguineous marriage. It is also concluded from the study that meiotic non disjunction is the commonest genotypic variation among the new-borns of DS than mosaicism. Nevertheless, given a family history of repeated miscarriages, the likelihood and significance of a chromosomal translocations in one of the parents cannot be understated.^10^ Early detection of trisomy 21 is important for a better counselling and management of the upcoming pregnancies. Over the past few decades, antenatal screening methods have evolved significantly comprising both invasive and non-invasive methods. Over the years, various screening strategies have been applied, and women are subjected to screen non-invasively for the chromosomal abnormalities in their foetuses if are ≥ 35 years of age, having any abnormal findings during first or second trimester ultrasound, or abnormal PAPP-A or free β-hCG levels during first or second trimester of pregnancy (Table-I). However these non-invasive methods only provide the risk of having an affected pregnancy, the definitive diagnosis is established with karyotyping of cultured fetal cells obtained by one of the invasive procedures such as chorionic villus sampling and amniocentesis. Of all the prenatal karyotyping tests performed the detection rate of trisomy 21 is approximately 1.6 – 3.2%.^11^ Furthermore, NIPT, or non-invasive prenatal testing, has gained widespread acceptance as a DS screening method. Using this technique, foetal DNA in mother plasma is analysed. With high accuracy results, it is currently being given as a substitute for intrusive testing to all high-risk cases.^10^ Nevertheless, it is important to note that our study lacks detailed information on these antenatal screening methods and criteria, which should be considered a limitation in our analysis.

**Table-I:** Risk Factors and Management of Children with Down syndrome.

Risk Factors Associated with DS	Screening for DS	Further Screening	Genetic Diagnostic Procedures	Management of DS
Advanced maternal age ≥ 35 years	Anomaly scan for congenital heart defect and abdominal wall defect	Non Invasive Prenatal Testing (NIPT)	Chorionic Villus Sampling (CVS) (11-14 weeks of gestation)	Physical, occupational and speech therapy
Abnormal U/S findings i.e. intrauterine growth restriction, increased nuchal fold thickness				Possible complications include heart defects, hearing and vision loss, celiac disease, breathing difficulties, sleep apnea, asthma, underactive thyroid, seizures, leukemia and early onset dementia
	Alpha- fetoprotein		Amniocentesis > 15 weeks of gestation	
	Beta-hCG			
	PAPP-A			

## CONCLUSION

The frequency of down syndrome in our study is slightly higher than the incidence estimated with in South East Asia. Further large-scale studies will be required in order to strengthen our findings. It is high time to perform effective antenatal screening and efficient prenatal diagnostic services for early detection of chromosomal numerical aberration such as Down syndrome for better assessment of the pregnancy and counselling of the couple for informed decision making, while keeping in mind the religious and cultural sentiments.

### Author contribution:

**AA:** Contributed to the study design, questionnaire design, data interpretation, and manuscript writing.

**NA:** Contributed to the study concept, study design, literature search, data collection, analysis, and interpretation.

**MIH:** Contributed to the literature search and provided feedback through critical manuscript review.

**SRA:** Provided feedback through critical manuscript review, and apprved the final version for publication

All authors have read the final version and are responsible and accountable for the accuracy and integrity of the work.
